# 2-(3,4-Dimeth­oxy­phen­yl)-1*H*-benzimidazole

**DOI:** 10.1107/S1600536811046897

**Published:** 2011-11-09

**Authors:** Aliakbar Dehno Khalaji, Alireza Foroghnia, Mohammad Ali Khalilzadeh, Karla Fejfarová, Michal Dušek

**Affiliations:** a Department of Chemistry, Faculty of Science, Golestan University, Gorgan, Iran; b Department of Chemistry, Islamic Azad University, Qaemshahr, Iran; c Institute of Physics of the ASCR, v.v.i, Na Slovance 2, 182 21 Praha 8, Czech Republic

## Abstract

In title compound, C_15_H_14_N_2_O_2_, the dihedral angle between the 3,4-dimeth­oxy­phenyl group and the benzimidazole system is 26.47 (6)°. In the crystal, neighbouring mol­ecules are linked by N—H⋯N hydrogen bonds into *C*(4) chains propagating along the *c*-axis direction. The crystal structure also features weak C—H⋯O inter­actions.

## Related literature

For a related structure, further synthetic details and background references to imidazolines, see: Khalaji *et al.* (2008 ▶). For related structures, see: Kia *et al.* (2008 ▶, 2009 ▶); Rashid *et al.* (2007 ▶).
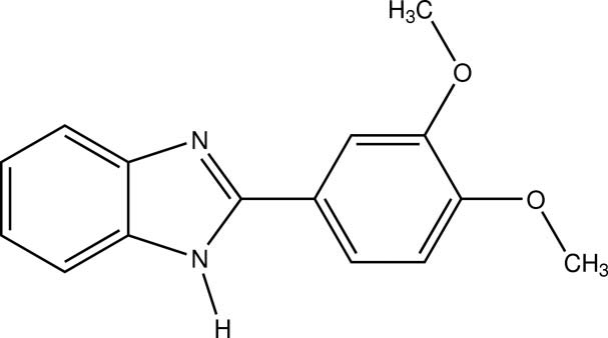


## Experimental

### 

#### Crystal data

C_15_H_14_N_2_O_2_
*M* *_r_* = 254.3
Orthorhombic, 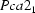

*a* = 9.2274 (8) Å
*b* = 15.0109 (9) Å
*c* = 9.2681 (3) Å
*V* = 1283.74 (14) Å^3^
*Z* = 4
Cu *K*α radiation
μ = 0.72 mm^−1^
*T* = 120 K
0.40 × 0.21 × 0.09 mm

#### Data collection

Agilent Xcalibur diffractometer with an Atlas (Gemini ultra Cu) detector
Absorption correction: multi-scan (*CrysAlis PRO*; Agilent, 2010 ▶) *T*_min_ = 0.75, *T*_max_ = 1
12506 measured reflections
1982 independent reflections
1919 reflections with *I* > 3σ(*I*)
*R*_int_ = 0.024

#### Refinement

*R*[*F* ^2^ > 3σ(*F* ^2^)] = 0.026
*wR*(*F* ^2^) = 0.072
*S* = 1.46
1982 reflections
174 parameters
H atoms treated by a mixture of independent and constrained refinement
Δρ_max_ = 0.08 e Å^−3^
Δρ_min_ = −0.11 e Å^−3^

### 

Data collection: *CrysAlis PRO* (Agilent, 2010 ▶); cell refinement: *CrysAlis PRO*; data reduction: *CrysAlis PRO*; program(s) used to solve structure: *SIR2002* (Burla *et al.*, 2003 ▶); program(s) used to refine structure: *JANA2006* (Petříček *et al.*, 2006 ▶); molecular graphics: *DIAMOND* (Brandenburg & Putz, 2005 ▶); software used to prepare material for publication: *JANA2006*.

## Supplementary Material

Crystal structure: contains datablock(s) global, I. DOI: 10.1107/S1600536811046897/hb6490sup1.cif

Structure factors: contains datablock(s) I. DOI: 10.1107/S1600536811046897/hb6490Isup2.hkl

Supplementary material file. DOI: 10.1107/S1600536811046897/hb6490Isup3.cml

Additional supplementary materials:  crystallographic information; 3D view; checkCIF report

## Figures and Tables

**Table 1:** Hydrogen-bond geometry (Å, °)

*D*—H⋯*A*	*D*—H	H⋯*A*	*D*⋯*A*	*D*—H⋯*A*
C14—H14*c*⋯O2^i^	0.96	2.49	3.224 (2)	133
C15—H15*b*⋯O1^ii^	0.96	2.50	3.372 (2)	151
N1—H1⋯N2^iii^	0.903 (19)	2.01 (2)	2.887 (2)	164.6 (16)

Symmetry codes: (i) 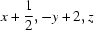; (ii) 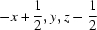; (iii) 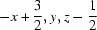.

**Fig. 1.**
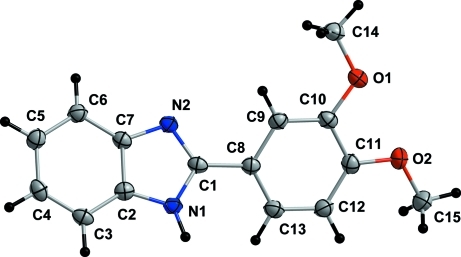
The structure of the title compound. Displacement ellipsoids are drawn at the 50% probability level.

**Fig. 2.**
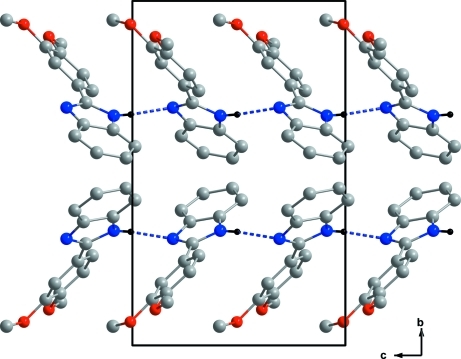
The crystal packing of the title compound, viewed along the a axis. Hydrogen bonds are drawn as dashed lines. Hydrogen atoms not participating in hydrogen bonds were omitted for clarity.

## supplementary crystallographic information

### Comment

As part of our ongoing studies of imidazoline derivatives (Khalaji *et al.*, 2008), we now report the synthesis and structure of the title compound, (I).

The dihedral angle between the N1/N2/C1—C7 and C8—C13 aromatic ring planes in (I) is 26.47 (6)°, which is comparable with related structures (Khalaji *et al.*, 2008; Kia *et al.*, 2008, 2009; Rashid *et al.*, 2007). Atoms C14 and C15 in (I) are displaced from the mean plane of the C8—C13 ring by 0.1134 (17) Å and 0.1756 (18) Å, respectively.

In the crystal of (I), an N—H···N hydrogen bond links the molecules into chains propagating in [001] direction (Fig. 2). There are no aromatic π-π stacking interactions in (I) as the closest centroid-centroid separation of aromatic rings is 4.419 (1) Å, which contrasts with the situation in 2-(4-fluorophenyl)-1*H*-benzimidazole (Rashid *et al.*, 2007) in which both N—H···N and π-π stacking help to establish the packing. The crystal structure of (I) is further stabilized by weak C—H···O interactions.

### Experimental

The synthetic method used for the preparation of (I) was based on previous work (Khalaji *et al.*, 2008), except that 3,4-dimethoxybenzaldehyde (2 mmol) was used.  Light yellow slabs of (I) were obtained by evaporation of a methanol solution of (I) held at room temperature. Anal. Calc. for C_15_H_14_N_2_O_2_ (MW: 254.30): C, 70.85; H, 5.55; N, 11.01%. Found: C, 70.92; H, 5.65; N, 11.08%. Yield: 73%. IR (KBr pellet, cm-1): 2941, 2977, 3006 (CH aliphatic and aromatic), 3054 (s, –C—HN–), 1624 (s, C=N), 1504, 1588, 1606 (C—C aromatic). ^1^H-NMR (500 MHz, CDCl3, δ(p.p.m.)): 3.82 (s, 3H), 3.87 (s, 3H), 7.11 (d, 1H), 7.17 (dd, 2H), 7.57 (s, 2H), 7.57–7.79 (m, 2H), 12.78 (s, 1H).

### Refinement

All hydrogen atoms were discernible in difference Fourier maps and could be refined to reasonable geometry. According to common practice they were nevertheless kept in ideal positions with C–H distance 0.96 Å during the refinement. The isotropic atomic displacement parameters of hydrogen atoms were evaluated as 1.5×*U*_eq_(C) for methyl groups and 1.2×*U*_eq_(C, N) for all other hydrogen atoms.

### Crystal data

**Table:**

C_15_H_14_N_2_O_2_	*F*(000) = 536
*M**_r_* = 254.3	*D*_x_ = 1.315 Mg m^−^^3^
Orthorhombic, *P**c**a*2_1_	Cu *K*α radiation, λ = 1.5418 Å
Hall symbol: P 2c -2ac	Cell parameters from 8286 reflections
*a* = 9.2274 (8) Å	θ = 2.9–67°
*b* = 15.0109 (9) Å	µ = 0.72 mm^−^^1^
*c* = 9.2681 (3) Å	*T* = 120 K
*V* = 1283.74 (14) Å^3^	Slab, light yellow
*Z* = 4	0.40 × 0.21 × 0.09 mm

### Data collection

**Table:**

Agilent Xcalibur diffractometer with an Atlas (Gemini ultra Cu) detector	1982 independent reflections
Radiation source: Enhance Ultra (Cu) X-ray Source	1919 reflections with *I* > 3σ(*I*)
mirror	*R*_int_ = 0.024
Detector resolution: 10.3784 pixels mm^-1^	θ_max_ = 67.1°, θ_min_ = 2.9°
Rotation method data acquisition using ω scans	*h* = −11→11
Absorption correction: multi-scan (*CrysAlis PRO*; Agilent, 2010)	*k* = −17→17
*T*_min_ = 0.75, *T*_max_ = 1	*l* = −9→11
12506 measured reflections	

### Refinement

**Table:**

Refinement on *F*^2^	54 constraints
*R*[*F*^2^ > 2σ(*F*^2^)] = 0.026	H atoms treated by a mixture of independent and constrained refinement
*wR*(*F*^2^) = 0.072	Weighting scheme based on measured s.u.'s *w* = 1/[σ^2^(*I*) + 0.0016*I*^2^]
*S* = 1.46	(Δ/σ)_max_ = 0.047
1982 reflections	Δρ_max_ = 0.08 e Å^−^^3^
174 parameters	Δρ_min_ = −0.11 e Å^−^^3^
0 restraints	

### Special details

**Table:**

Experimental. CrysAlisPro, Agilent Technologies (2010), Empirical absorption correction using spherical harmonics, implemented in SCALE3 ABSPACK scaling algorithm.
Refinement. The refinement was carried out against all reflections. The conventional *R*-factor is always based on *F*. The goodness of fit as well as the weighted *R*-factor are based on *F* and *F*^2^ for refinement carried out on *F* and *F*^2^, respectively. The threshold expression is used only for calculating *R*-factors *etc*. and it is not relevant to the choice of reflections for refinement.The program used for refinement, Jana2006, uses the weighting scheme based on the experimental expectations, see _refine_ls_weighting_details, that does not force *S* to be one. Therefore the values of *S* are usually larger than the ones from the *SHELX* program.

### Fractional atomic coordinates and isotropic or equivalent isotropic displacement parameters (Å^2^)

**Table:**

	*x*	*y*	*z*	*U*_iso_*/*U*_eq_	
O1	0.50788 (10)	0.93193 (6)	0.50931 (11)	0.0289 (3)	
O2	0.26137 (11)	0.89618 (7)	0.39223 (16)	0.0325 (3)	
N1	0.77384 (12)	0.66702 (8)	0.08732 (18)	0.0233 (3)	
N2	0.86211 (11)	0.69056 (8)	0.30939 (17)	0.0238 (3)	
C1	0.75266 (14)	0.70471 (9)	0.21870 (18)	0.0226 (4)	
C2	0.90804 (14)	0.62618 (9)	0.09065 (19)	0.0228 (4)	
C3	0.98463 (15)	0.57777 (9)	−0.01205 (19)	0.0264 (4)	
C4	1.11744 (15)	0.54402 (10)	0.0297 (2)	0.0281 (4)	
C5	1.17424 (16)	0.55964 (9)	0.1677 (2)	0.0285 (4)	
C6	1.09725 (15)	0.60805 (9)	0.2701 (2)	0.0268 (4)	
C7	0.96186 (14)	0.64097 (9)	0.2299 (2)	0.0228 (4)	
C8	0.62258 (13)	0.75563 (9)	0.25552 (18)	0.0238 (4)	
C9	0.63170 (14)	0.82035 (9)	0.36407 (19)	0.0238 (4)	
C10	0.51002 (15)	0.86699 (9)	0.4062 (2)	0.0244 (4)	
C11	0.37543 (14)	0.84855 (10)	0.34023 (19)	0.0261 (4)	
C12	0.36736 (14)	0.78589 (11)	0.2312 (2)	0.0293 (4)	
C13	0.49052 (15)	0.73941 (10)	0.1886 (2)	0.0277 (4)	
C14	0.63841 (15)	0.94452 (11)	0.5909 (2)	0.0311 (4)	
C15	0.12101 (16)	0.87431 (12)	0.3367 (2)	0.0413 (5)	
H3	0.947018	0.568253	−0.107386	0.0316*	
H4	1.172283	0.508955	−0.037558	0.0337*	
H5	1.268095	0.53647	0.191941	0.0342*	
H6	1.135836	0.618397	0.364821	0.0322*	
H9	0.723198	0.832316	0.409397	0.0286*	
H12	0.276306	0.774417	0.184648	0.0352*	
H13	0.48424	0.696024	0.112722	0.0333*	
H14a	0.62263	0.989168	0.663353	0.0467*	
H14b	0.665129	0.889441	0.636306	0.0467*	
H14c	0.714823	0.963371	0.527559	0.0467*	
H15a	0.049359	0.91136	0.381853	0.062*	
H15b	0.119532	0.883995	0.234322	0.062*	
H15c	0.10002	0.812894	0.356683	0.062*	
H1	0.7167 (18)	0.6702 (11)	0.008 (2)	0.028*	

### Atomic displacement parameters (Å^2^)

**Table:**

	*U*^11^	*U*^22^	*U*^33^	*U*^12^	*U*^13^	*U*^23^
O1	0.0310 (5)	0.0286 (5)	0.0270 (6)	0.0021 (4)	−0.0007 (4)	−0.0067 (4)
O2	0.0253 (5)	0.0362 (6)	0.0359 (6)	0.0074 (4)	−0.0002 (4)	−0.0064 (5)
N1	0.0238 (5)	0.0285 (6)	0.0176 (6)	0.0003 (4)	−0.0005 (5)	−0.0009 (5)
N2	0.0239 (5)	0.0289 (6)	0.0186 (6)	0.0017 (4)	0.0007 (4)	−0.0010 (5)
C1	0.0249 (6)	0.0252 (6)	0.0178 (6)	−0.0029 (5)	0.0022 (5)	0.0003 (5)
C2	0.0240 (6)	0.0233 (6)	0.0209 (7)	−0.0025 (5)	0.0012 (6)	0.0026 (5)
C3	0.0316 (7)	0.0282 (7)	0.0193 (7)	−0.0032 (6)	0.0030 (5)	−0.0011 (5)
C4	0.0299 (7)	0.0274 (7)	0.0268 (7)	0.0015 (5)	0.0081 (6)	−0.0009 (6)
C5	0.0260 (7)	0.0315 (7)	0.0281 (7)	0.0037 (6)	0.0017 (6)	0.0022 (6)
C6	0.0262 (6)	0.0319 (7)	0.0224 (8)	0.0003 (5)	−0.0016 (5)	−0.0005 (6)
C7	0.0241 (6)	0.0250 (7)	0.0193 (7)	−0.0016 (5)	0.0035 (6)	0.0004 (5)
C8	0.0255 (7)	0.0255 (7)	0.0205 (7)	0.0008 (5)	0.0008 (5)	0.0014 (5)
C9	0.0247 (6)	0.0264 (7)	0.0204 (7)	−0.0004 (5)	−0.0001 (5)	0.0020 (6)
C10	0.0304 (7)	0.0236 (7)	0.0193 (7)	−0.0002 (5)	0.0023 (5)	0.0015 (6)
C11	0.0265 (7)	0.0280 (7)	0.0238 (7)	0.0028 (5)	0.0033 (5)	0.0019 (6)
C12	0.0259 (7)	0.0362 (8)	0.0258 (8)	0.0018 (5)	−0.0037 (6)	−0.0014 (6)
C13	0.0303 (7)	0.0314 (7)	0.0215 (7)	−0.0001 (6)	−0.0013 (5)	−0.0030 (6)
C14	0.0336 (7)	0.0308 (8)	0.0291 (8)	−0.0019 (5)	−0.0021 (6)	−0.0070 (6)
C15	0.0282 (8)	0.0531 (10)	0.0428 (11)	0.0092 (7)	−0.0067 (7)	−0.0144 (8)

### Geometric parameters (Å, °)

**Table:**

O1—C10	1.3651 (18)	C6—C7	1.3942 (19)
O1—C14	1.4345 (18)	C6—H6	0.96
O2—C11	1.3606 (18)	C8—C9	1.401 (2)
O2—C15	1.4319 (19)	C8—C13	1.389 (2)
N1—C1	1.357 (2)	C9—C10	1.380 (2)
N1—C2	1.3820 (17)	C9—H9	0.96
N1—H1	0.903 (19)	C10—C11	1.412 (2)
N2—C1	1.331 (2)	C11—C12	1.383 (2)
N2—C7	1.3943 (19)	C12—C13	1.391 (2)
C1—C8	1.4633 (19)	C12—H12	0.96
C2—C3	1.391 (2)	C13—H13	0.96
C2—C7	1.401 (2)	C14—H14a	0.96
C3—C4	1.381 (2)	C14—H14b	0.96
C3—H3	0.96	C14—H14c	0.96
C4—C5	1.402 (3)	C15—H15a	0.96
C4—H4	0.96	C15—H15b	0.96
C5—C6	1.391 (2)	C15—H15c	0.96
C5—H5	0.96		
			
C10—O1—C14	116.79 (11)	C9—C8—C13	119.67 (12)
C11—O2—C15	116.86 (13)	C8—C9—C10	120.42 (13)
C1—N1—C2	107.09 (13)	C8—C9—H9	119.7897
C1—N1—H1	128.4 (11)	C10—C9—H9	119.7883
C2—N1—H1	124.4 (11)	O1—C10—C9	124.92 (13)
C1—N2—C7	104.62 (14)	O1—C10—C11	115.50 (12)
N1—C1—N2	113.01 (12)	C9—C10—C11	119.59 (15)
N1—C1—C8	123.04 (13)	O2—C11—C10	115.09 (14)
N2—C1—C8	123.95 (15)	O2—C11—C12	125.08 (13)
N1—C2—C3	132.21 (16)	C10—C11—C12	119.83 (13)
N1—C2—C7	105.55 (13)	C11—C12—C13	120.32 (13)
C3—C2—C7	122.23 (13)	C11—C12—H12	119.839
C2—C3—C4	116.80 (16)	C13—C12—H12	119.8382
C2—C3—H3	121.5999	C8—C13—C12	120.14 (15)
C4—C3—H3	121.5996	C8—C13—H13	119.9308
C3—C4—C5	121.72 (15)	C12—C13—H13	119.9316
C3—C4—H4	119.1383	O1—C14—H14a	109.4704
C5—C4—H4	119.138	O1—C14—H14b	109.4712
C4—C5—C6	121.28 (14)	O1—C14—H14c	109.4714
C4—C5—H5	119.362	H14a—C14—H14b	109.4712
C6—C5—H5	119.3623	H14a—C14—H14c	109.471
C5—C6—C7	117.42 (16)	H14b—C14—H14c	109.4722
C5—C6—H6	121.2877	O2—C15—H15a	109.4714
C7—C6—H6	121.2879	O2—C15—H15b	109.4715
N2—C7—C2	109.72 (12)	O2—C15—H15c	109.4712
N2—C7—C6	129.76 (16)	H15a—C15—H15b	109.4715
C2—C7—C6	120.52 (14)	H15a—C15—H15c	109.4712
C1—C8—C9	118.71 (12)	H15b—C15—H15c	109.4705
C1—C8—C13	121.59 (14)		
			
C9—C10—O1—C14	−7.8 (2)	N1—C1—C8—C9	−154.38 (14)
C10—C11—O2—C15	−174.97 (15)		

### Hydrogen-bond geometry (Å, °)

**Table:**

*D*—H···*A*	*D*—H	H···*A*	*D*···*A*	*D*—H···*A*
C14—H14c···O2^i^	0.96	2.49	3.224 (2)	133
C15—H15b···O1^ii^	0.96	2.50	3.372 (2)	151
N1—H1···N2^iii^	0.903 (19)	2.01 (2)	2.887 (2)	164.6 (16)

Symmetry codes: (i) *x*+1/2, −*y*+2, *z*; (ii) −*x*+1/2, *y*, *z*−1/2; (iii) −*x*+3/2, *y*, *z*−1/2.
